# Evidence of innate immune dysfunction in first-episode psychosis patients with accompanying mood disorder

**DOI:** 10.1186/s12974-022-02648-y

**Published:** 2022-12-03

**Authors:** Heather K. Hughes, Houa Yang, Tyler A. Lesh, Cameron S. Carter, Paul Ashwood

**Affiliations:** 1 Department of Medical Microbiology and Immunology, University of California at Davis, Davis, CA 95616 USA; 2 MIND Institute, University of California at Davis, 2805, 50Th Street, Sacramento, CA 95817 USA; 3 Department of Psychiatry and Behavioral Sciences, University of California at Davis, Davis, CA 95616 USA

**Keywords:** Psychosis, Schizophrenia, Bipolar disorder, Major depressive disorder, Affective, Autism, Immune, Cytokine, Inflammation, Neurodevelopmental disorders

## Abstract

**Background:**

Inflammation and increases in inflammatory cytokines are common findings in psychiatric disorders such as schizophrenia (SCZ), bipolar disorder (BD), and major depressive disorder (MDD). Meta-analyses of studies that measured circulating cytokines have provided evidence of innate inflammation across all three disorders, with some overlap of inflammatory cytokines such as IL-6 and TNF-α. However, differences across disorders were also identified, including increased IL-4 in BD that suggest different immune mechanisms may be involved depending on the type of disorder present.

**Methods:**

We sought to identify if the presence or absence of an affective disorder in first-episode psychotic (FEP) patients was associated with variations in cytokine production after stimulation of peripheral blood mononuclear cells (PBMC). 98 participants were recruited and grouped into healthy controls (*n* = 45) and first-episode psychosis patients (*n* = 53). Psychosis patients were further grouped by presence (AFF; *n* = 22) or lack (NON; *n* = 31) of an affective disorder. We cultured isolated PBMC from all participants for 48 h at 37 °C under four separate conditions; (1) culture media alone for baseline, or the following three stimulatory conditions: (2) 25 ng/mL lipopolysaccharide (LPS), (3) 10 ng/mL phytohemagglutinin (PHA), and (4) 125 ng/ml α-CD3 plus 250 ng/ml α-CD28. Supernatants collected at 48 h were analyzed using multiplex Luminex assay to identify differences in cytokine and chemokine production. Results from these assays were then correlated to patient clinical assessments for positive and negative symptoms common to psychotic disorders.

**Results:**

We found that PBMC from affective FEP patients produced higher concentrations of cytokines associated with both innate and adaptive immunity after stimulation than non-affective FEP patients and healthy controls. More specifically, the AFF PBMC produced increased tumor necrosis fctor (TNF)-α, interleukin (IL)-1β, IL-6, and others associated with innate inflammation. PBMC from AFF also produced increased IL-4, IL-17, interferon (IFN)γ, and other cytokines associated with adaptive immune activation, depending on stimulation. Additionally, inflammatory cytokines that differed at rest and after LPS stimulation correlated with Scale for the Assessment of Negative Symptoms (SANS) scores.

**Conclusions:**

Our findings suggest that immune dysfunction in affective psychosis may differ from that of primary psychotic disorders, and inflammation may be associated with increased negative symptoms. These findings could be helpful in determining clinical diagnosis after first psychotic episode.

**Supplementary Information:**

The online version contains supplementary material available at 10.1186/s12974-022-02648-y.

## Background

Affective and psychotic disorders such as schizophrenia (SCZ), bipolar disorder (BD), and major depressive disorder (MDD) are psychiatric disorders that are responsible for significant morbidity and make a substantial contribution to disease burden [1–3]. The etiology of these disorders is complex and elusive; however, there is accumulating evidence that immune dysfunction may be contributing to the pathogenesis of these disorders [4]. Maternal infection, familial autoimmunity, and immune-mediated diseases such as asthma are risk factors for affective and psychotic disorders [5–8]. Severe infections, especially those requiring hospitalization and those treated with antibiotics, are also risk factors for these disorders, which could suggest these disorders are being mediated by inflammatory responses or changes in the microbiota [9, 10].

A role for the immune system in individuals with these disorders has been proposed for decades, with an emphasis on inflammatory cytokine production associated with chronically activated macrophages and T cells mediating symptoms of psychosis [11, 12]. Elevated blood cytokine levels associated with innate immunity have been identified repeatedly in SCZ, BD, and MDD [13–18], including a recent meta-analysis that showed overlap of inflammatory cytokines across all three disorders [19]. Consistently elevated cytokines included pro-inflammatory interleukin (IL)-6 and tumor necrosis factor (TNF)-α, both of which are associated with classical innate immune activation and play a critical role in acute phase response during early infection [20, 21]. IL-1β, soluble IL-2 receptor (sIL-2R), IL-17, soluble TNF receptor type 1 (sTNF-R1), and IL-1RA are among other frequently elevated cytokines reported in meta-analyses of these disorders [14–17, 19, 22, 23]. Although circulating cytokines provide some information about the inflammatory status of an individual at the time they are enrolled in the study, results are often heterogenous and do not provide evidence as to which immune cells are dysfunctional or aberrantly respond to stimulation.

Cellular studies that identify differences in cell populations and cellular activity have provided more insight into the immune dysfunction present in these disorders. Increases in circulating monocytes and T cells have been noted in these disorders [24–28], often with increases in inflammatory gene and protein expression (29–32). In BD, increased production of TNF-α and IL-6 in manic patients was seen after phytohemagglutinin (PHA) stimulation of whole blood, while IL-4 was decreased compared to controls [33]. Impaired T cell and NK cell subsets were also identified in MDD, with regulatory T cell deficiencies in older MDD patients associated with inflammatory activation of monocytes [34]. Findings of elevated IL-1β, IL-6, and IL-8 in cerebrospinal fluid suggest that the increased inflammation in these disorders is not only peripheral but may include neuroinflammation [35]. Evidence of inflammation within the brain has also been noted through post-mortem and positron emission tomography (PET) studies, within increased radioligand binding to mitochondrial translocator protein 18 kDa, a marker of microglial activation. However, data are conflicting in these studies and some studies point to reduced microglia activation in these disorders [36]. Although these findings are varied and no consensus has been reached due to these inconsistencies, inflammation was often present based on state of disease (reviewed in [37]). For example, major affective episodes have been associated with neuroinflammation, including evidence of microglia activation in suicidal patients [38–41]. Paranoid episodes of SCZ have also been linked to neuroinflammation and microglia activation [42].

FEP patients typically receive a diagnosis of schizophrenia-spectrum psychosis or an affective disorder with psychotic features. Differentiating between these can be challenging in the early diagnostic process, as FEP patients are subject to fluctuating symptoms that may lead to future changes in diagnosis [43]. Additional exploration of immune differences between affective and non-affective psychotic disorders could identify risk factors and biomarkers that might be helpful in accurate diagnosis at presentation with the first episode. For example, analysis of a large Danish cohort found that familial autoimmune disorders are a risk factor for SCZ with non-affective psychosis but not bipolar disorder, providing some evidence of separate immune-associated risks in these disorders [44]. A previous study identified increases in serum cytokines associated with innate immune activation in FEP when compared to healthy controls; however, TNF-α and IL-4 were elevated further in FEP patients with depression compared to those without depression [45]. We recently compared plasma cytokines in FEP patients diagnosed with SCZ or BD. Elevated inflammatory cytokines were seen in plasma of SCZ patients, while analysis of plasma from BD patients revealed increased IL-10 [18]. We later found dampened macrophage activity after inflammatory stimulation in non-affective psychosis patients compared to those with affective disorder and healthy controls. However, affective psychosis patients exhibited increased production of pro-inflammatory cytokines after alternative activation of macrophages [46]. Taken together, these findings suggest there might be differences in the immune status of FEP patients based on presence or absence of an affective disorder. With this in mind, we sought to determine if cytokine production of stimulated peripheral blood mononuclear cells (PBMC) varies significantly in affective compared to non-affective psychosis patients and healthy controls to help further delineate immune dysfunction across these disorders.

## Methods and materials

### Study participants

We recruited 98 participants to our study, including 45 healthy controls and 53 first-episode psychosis patients who were further categorized based on presence or absence of an affective disorder which included bipolar disorder and major depressive disorder with psychotic features (AFF group), or schizophrenia-spectrum diagnosis (NON group). Participants were between the ages of 13 and 37 years and were clinically assessed using the Structured Clinical Interview for the DSM-IV-TR SCID-I/P (demographic information in Table 1). Clinical ratings were collected in the patient sample using the Scale for the Assessment of Negative Symptoms (SANS; [47]), Scale for the Assessment of Positive Symptoms (SAPS; [48]), and Global Assessment of Functioning (GAF; [49]). The majority of patients were taking antipsychotic medications, which were converted to chlorpromazine (CPZ) equivalent dose to assess relative antipsychotic potencies. Participants were excluded for positive urine toxicology at the time of testing, alcohol or drug abuse/dependence within three months of assessment, and/or a Wechsler Abbreviated Scale of Intelligence (WASI) IQ score that was below 70. In addition, HC were excluded for presence of any Axis I or Axis II disorder or psychotic disorder within first-degree family members. Diagnoses were later confirmed at a 12-month assessment. The University of California, Davis Institutional Review Board approved this study.

**Table 1:** Demographic characteristics and clinical scores

	HC (*n* = 45)	AFF (*n* = 22)	NON (*n* = 31)	*P* values		
				HC vs AFF	AFF vs NON	HC vs NON
Age: median	20.7	22.0	21.1	0.3141	> 0.9999	> 0.9999
Range [minimum, maximum]	[13.7, 37.0]	[13.2, 29.6]	[13.3, 26.8]			
IQR	18.3–22.7	19.4–26.1	19.6–22.8			
Gender (%male/female)	62/38	71/29	84/16	0.4280	0.4933	0.0702
Subject education: median (IQR)	14 (12–15)	12.5 (12–14.8)	12 (11–13.3)	> 0.9999	0.7141	0.0638
Parental education: median (IQR)	15 (13–16)	14 (12–17)	15 (12.3–16)	> 0.9999	> 0.9999	> 0.9999
SANS: median (IQR)	–	7 (3–8)	10 (6.3–12)	–	*0.0191*	
SAPS: median (IQR)	–	2 (1–4.8)	6 (3–8)	–	*0.0311*	–
WASI: median (IQR)	117 (110–120)	109 (89–116)	99 (89.5–106)	*0.003*	> 0.9999	< *0.0001*
GAF: median (IQR)	88 (80–90)	48 (40–68)	43.5 (37.8–54.5)	< *0.0001*	0.9874	< *0.0001*
Antipsychotic dose: CPZ mg median (range)	–	200 (67–1000)	200 (50–909)	–	0.2879	–

*CTL* control, *AFF* affective, *NON* non-affective, *IQR* interquartile range, *SANS* Scale for the Assessment of Negative Symptoms, *SAPS* Scale for the Assessment of Positive Symptoms, *WASI* Wechler Abbreviated Scale of Intelligence, *GAF* Global Assessment of Functioning, *CPZ* chlorpromazine equivalent dose

### Cell isolation

Peripheral blood was collected from each participant in an acid–citrate–dextrose Vacutainer tube (BD Biosciences, San Jose, California) and processed within 12 h of collection. The blood was centrifuged for 10 min at 2100 rpm, then plasma was removed. The remaining blood was diluted 1:2 with Hanks Balanced Salt Solution (HBSS; Gibco, Gaithersburg, MD) then carefully layered over a Ficoll-Paque gradient (Pharmacia Biotech, Piscataway, NJ) and centrifuged at 1700 rpm for 30 min at room temperature. Peripheral blood mononuclear cells (PBMC) were collected from the interface layer and washed twice with HBSS. Cell viability was determined by trypan blue exclusion. The cells were resuspended at a final concentration of 3 × 10^6^ cells/mL in a solution of 0.2% T-Stim (BD Biosciences) in X-Vivo media (Cambrex, Walkersville, MD).

### Cell stimulation

Freshly collected PBMC were plated in X-Vivo media at 3 × 10^5^ cells/well and cultured at 37 °C with 5% CO_2_. The cells were either stimulated alone (no treatment), or with 25 ng/mL lipopolysaccharide (LPS; Sigma-Aldrich; St. Louis, MO) to activate Toll-like receptor (TLR)-4, 10 ng/mL phytohemagglutinin (PHA; Sigma-Aldrich; St. Louis, MO) to broadly activate T and B cells (adaptive immunity), or 125 ng/mL α-CD3 plus 250 ng/mL α-CD28 (α-CD3/CD28; Sigma-Aldrich; St. Louis, MO) to specifically co-stimulate and induce T cell adaptive immune responses. After 48 h, plates were centrifuged at 2100 rpm for 10 min, and supernatants were collected and stored at − 80 °C until analysis.

### Cytokine and chemokine analysis

To assess PBMC response to stimulation, supernatants from PBMC cultures were quantified via multiplex analysis using a high sensitivity Luminex bead set (Millipore, Saint Charles, MO) for innate inflammatory cytokines IL-1α, IL-1β, IL-6, IL-15 TNF-α,TNF-β, and interferon (IFN)-α2, inflammatory chemokines Eotaxin, IL-8, IFNγ inducible protein (IP-10/CXCL10), monocyte chemoattractant protein-1 (MCP-1/CCL2), and macrophage inflammatory protein 1-beta (MIP-1β/CCL4), T helper cell 1 (T_H_1)-associated cytokines IFNγ, IL-2, IL-12(p40), and IL-12(p70), T_H_2-associated cytokines IL-4, IL-5 and IL-13, the T_H_17 associated cytokine IL-17, anti-inflammatory/regulatory cytokines IL-10 and TGF-β. Granulocyte colony-stimulating factor (G-CSF), Granulocyte–macrophage colony-stimulating factor (GM-CSF) and IL-7 were measured to identify production of growth mediators. Cellular supernatants were incubated with antibody-coupled fluorescent beads for 2 h, then plates were washed. Biotinylated detection antibodies were then added to each well and incubated at room temperature for 1 h, followed by streptavidin–phycoerythrin and an additional 30-min incubation. The samples were analyzed using a flow-based Luminex™ 100 suspension array system (Bio-Plex 200; Bio-Rad Laboratories, Inc.). Unknown sample cytokine concentrations were calculated by Bio-Plex Manager software using a standard curve derived from the known reference cytokine standards provided in each kit. The sensitivity of this assay allowed the detection of cytokine concentrations with the following limits of detection: Eotaxin 4.0 pg/mL, G-CSF 1.8 pg/mL, GM-CSF 7.5 pg/mL, IFNα2 2.9 pg/mL, IFNγ 0.8 pg/mL, IL-1α 9.4 pg/mL, IL-1β 0.8 pg/mL, IL-2 1.0 pg/mL, IL-4 4.5 pg/mL, IL-5 0.5 pm/mL, IL-6 0.2 pg/mL, IL-7 1.4 pg/mL, IL-8 0.4 pg/mL, IL-10 0.48 pg/mL, IL-12p40 7.4 pg/mL, IL-12p70 0.6 pg/mL, IL-13 1.3 pg/mL, IL-15 1.2 pg/mL, IL-17 0.7 pg/mL, IP-10 8.6 pg/mL, MCP-1 1.9 pg/mL, MIP-1β 3.0 pg/mL, TGF-β pg/mL 12.0 pg/mL, TNF-α 0.7 pg/mL, and TNF-β 1.5 pg/mL. Values below the limit of detection (LOD) were replaced with one half the LOD.

### Statistical analysis

Shapiro–Wilk tests determined that the majority of the cytokine data were not normally distributed. Outliers were removed using ROUT with a coefficient Q set to 1%. Kruskal–Wallis tests were used to analyze differences across the three groups and Mann–Whitney U tests were then used for pairwise analyses, with the Holm–Sidak method used to correct for multiple comparisons. A two-tailed alpha of *p* < 0.05 was considered statistically significant. Behavioral correlations were calculated using Spearman’s Rho. Statistical analyses were carried out using analyses software GraphPad Prism v9.3e (GraphPad Software, San Diego, CA) and SPSS Statistics Version 28 (IBM, Armonk, NY, United States).

## Results

### Demographic and clinical characteristics

Participant demographics and clinical measures are summarized in Table 1. Participants ranged in age from 13 to 30 years old, and there were no differences in age between groups. There were more males in the NON group compared to the HC group; however, this did not reach statistical significance (Fisher’s exact test *p* = 0.0702). There were no significant differences in parent level of education across groups; however, among the participants the NON group level of education trended lower than HC (*p* = 0.0638). SANS (*p* = 0.0191) and SAPS (*p* = 0.0311) scores were both lower in the AFF group compared to the NON group. WASI scores were significantly lower in both the AFF group (*p* = 0.003) and the NON group (*p* < 0.0001) compared to the HC group. The HC group scored higher on GAF compared to both the AFF group (*p* < 0.0001) and the NON group (*p* < 0.0001). No significant differences were seen in dose of antipsychotic medication using chlorpromazine equivalent doses for estimation in AFF compared to NON.

### Baseline cytokine and chemokine production (media alone)

**Fig. 1:**
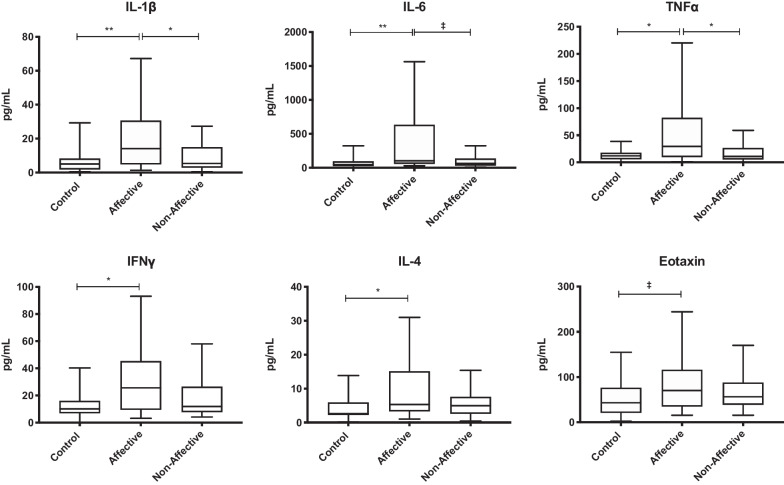
Basal cytokine and chemokine production in unstimulated PBMC culture supernatants following 48 h in media alone. Box and whiskers plots depict median, upper and lower interquartile ranges, *p < 0.05, **p < 0.01, ‡ indicates comparisons that were significant prior to multiple testing correction. HC *n* = 45, AFF *n* = 22, NON *n* = 31

To assess baseline production of cytokines, chemokines and growth factors from PBMC, we first analyzed supernatant after 48 h of culture in media alone. Kruskal–Wallis tests showed significant differences in IL-1β (*p* < 0.01), IL-6 (*p* < 0.01), TNF-α (*p* < 0.05), eotaxin (*p* < 0.05), IFNγ (*p* < 0.05), and IL-4 (*p* < 0.05) across the three groups. For pairwise comparisons, Mann–Whitney U tests revealed increased IL-1β (*p* = 0.0024), IL-6 (*p* = 0.0030), IFNγ (*p* = 0.0217), and IL-4 (*p* = 0.0241) in the AFF group compared to HC after statistical correction for multiple testing. Cell cultures from AFF participants also had increased IL-1β (p = 0.0329) and TNF-α (*p* = 0.0158) compared to the NON group (Table 2, Fig. 1).

**Table 2:** Concentration of cytokines/chemokines at baseline (media only)

		HC		AFF		NON	
		Median	(IQR)	Median	(IQR)	Median	(IQR)
Growth factors	G-CSF	6.3	(2.22–12.92)	13.95	(5.255–29.02)	10.43	(2.603–13.95)
	GM-CSF	16.96	(8.22–21.53)	19.46	(9.02–30.77)	18.5	(12.65–32.69)
	IL-7	12.81	(4.23–21)	19.04	(8.415–28.79)	18.04	(6.81–23.61)
Innate inflammatory cytokines	TNF-α	11.9	(5.65–17.79)	29.41*^#^	(9.778–82.13)	10.89	(5.28–26.76)
	TNF-β	1.14	(0.75–4.14)	3.050	(0.85–4.9)	2.455	(0.9–4.77)
	IL-1α	6.065	(4.7–22.28)	11.24	(4.85–20.54)	9.03	(3.89–22.65)
	IL-1β	5.03	(1.74–8.22)	14.12*^#^	(4.798–30.63)	5.33	(2.86–14.89)
	IL-6	45.95	(23.6–94.35)	102.7^**#**^	(55.69–632.4)	63.51	(31.24–135.2)
	IL-15	BLD	(–)	BLD	(–)	BLD	(–)
	IFNα2	26.47	(11.53–35.5)	19.7	(13.93–35.25)	26.51	(13.09–37.02)
Innate inflammatory chemokines	Eotaxin	43.02	(20.95–76.44)	70.46	(34.75–116)	56.27	(38.81–87.79)
	IL-8	10,401	(3718–16,599)	14,272	(9378–18,994)	10,897	(8047–18,622)
	IP-10	41.11	(13.06–77.82)	43.54	(14.26–62.98)	28.47	(18.58–111.9)
	MCP-1	10,124	(3185–13,321)	10,813	(9638–13,560)	9747	(3991–13,005)
	MIP-1β	69.72	(27.37–156.7)	100.5	(39.2–206.5)	88.83	(23.71–219.2)
T_H_1 cytokines	IFNγ	10.16	(6.87–16.02)	25.64^**#**^	(9.373–45.39)	11.86	(7.65–26.45)
	IL-2	0.52	(0.5–5.18)	1.71	(0.725–3.185)	1.325	(0.5–3.19)
	IL-12p40	6.57	(3.95–14.93)	12.12	(8.155–35.08)	11.3	(5.388–17.32)
	IL-12p70	2.19	(0.78–3.81)	3.98	(1.333–7.93)	2.19	(0.685–5.965)
T_H_2 cytokines	IL-4	2.62#	(2.25–5.923)	5.3^**#**^	(3.33–15.13)	4.955	(2.598–7.585)
	IL-5	BLD	( -)	BLD	( -)	BLD	( -)
	IL-13	2.4	(0.6675–5.015)	3.04	(1.885–5.4)	3.67	(1.03–5.97)
T_H_17 cytokine	IL-17	1.615	(0.425–5.158)	3.865	(1.47–6.898)	2.78	(1.505–5.975)
Regulatory cytokines	IL-10	6.77	(3.755–15.51)	21.13	(4.94–32.73)	6.77	(5.235–11.47)
	TGF-β	1732	(1040–2594)	2235	(1123–3029)	2059	(1304–2611)

*BLD* below the limit of detection

#*p* value < 0.05 when comparing AFF with HC

*p value < 0.05 when comparing AFF with NON

^†^*p* value < 0.05 when comparing NON with HC

### Cytokine and chemokine production after LPS stimulation

**Fig. 2:**
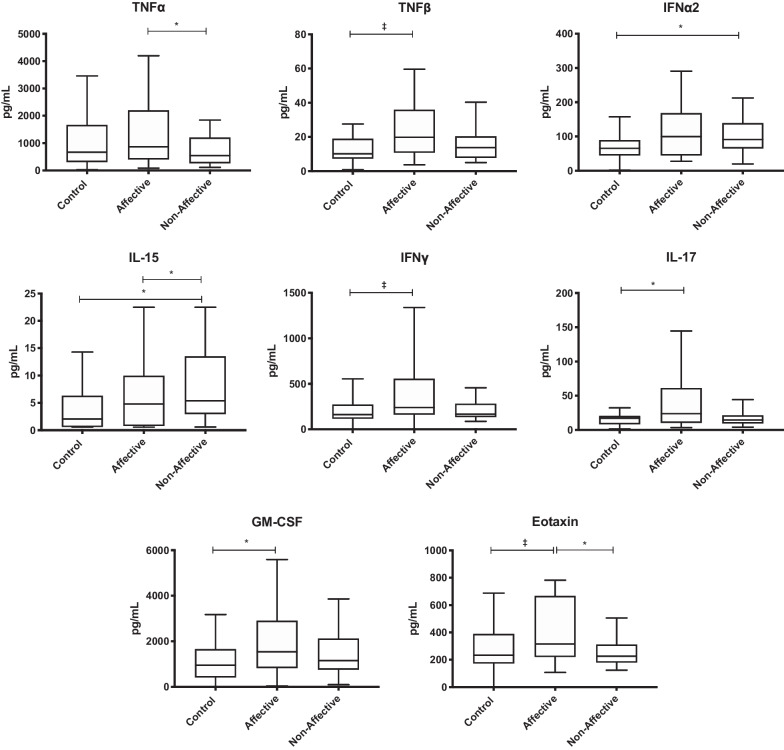
Cytokine and chemokine production from PBMC cell cultures following stimulation with LPS. Box and whiskers plots depict median, upper and lower interquartile ranges, *p < 0.05, ‡ indicates comparisons that were significant prior to multiple testing correction. HC *n* = 45, AFF *n* = 22, NON *n* = 31

To stimulate primarily innate immune responses, cell cultures were activated with LPS and supernatants assessed for cytokine production following LPS stimulation. Kruskal–Wallis tests revealed significant differences in eotaxin (*p* < 0.05), GM-CSF (*p* < 0.05), IL-15 (*p* < 0.05), IL-17 (*p* < 0.05) and IFNα2 (*p* < 0.01), with trending differences in IFNγ, TNF-α and TNF-β across the three groups following LPS stimulation. After statistical correction for multiple comparisons, Mann–Whitney *U* tests showed significantly increased levels of GM-CSF (*p* = 0.0288) and IL-17 (*p* = 0.0446) in the AFF group compared to HC. Eotaxin (*p* = 0.0446) and TNF-α (*p* = 0.0435) were also significantly increased in the AFF group compared to the NON group, IL-15 was increased uniquely in the NON group compared to both HC (*p* = 0.0344) and AFF (*p* = 0.0468) (Table 3, Fig. 2).

**Table 3:** Concentration of cytokines/chemokines after LPS stimulation

		HC		AFF		NON	
		Median	(IQR)	Median	(IQR)	Median	(IQR)
Growth factors	G-CSF	7004	(3616–10,607)	5839	(3855–11,409)	7026	(3560–10,522)
	GM-CSF	951.5	(411.9–1662)	2076^**#**^	(863.6–2998)	1157	(758.4–2120)
	IL-7	147.1	(97.3–220.9)	130.4	(91.93–253.3)	149.5	(81.36–219)
Innate inflammatory cytokines	TNF-α	667.3	(307.1–1665)	1081*	(475–2501)	549.8	(265.9–1206)
	TNF-β	10.07	(7.21–18.99)	18.430	(10.46–30.52)	13.73	(7.79–20.32)
	IL-1α	676.5	(417.6–931.3)	979.1	(375.7–1274)	621.2	(423.4–1001)
	IL-1β	872.3	(478.3–1708)	1884	(499.7–2849)	932.4	(499.4–1326)
	IL-6	24,378	(17,727–34,327)	25,326	(18,675–28,548)	22,498	(9904–28,025)
	IL-15	2.045	(0.6–6.293)	2.365	(0.6–5.453)	5.39*†	(2.958–13.53)
	IFNα2	65.52	(44.42–89.09)	93.99	(42.76–168.3)	91.28†	(65–139.3)
Innate inflammatory chemokines	Eotaxin	232.7	(172–388.3)	325.6*	(227.8–657.8)	226.6	(179.4–312.5)
	IL-8	28,706	(22,063–42,134)	32,535	(29,256–42,602)	29,426	(22,479–38,281)
	IP-10	123.6	(94.64–163.4)	173.7	(103.4–299.4)	117.2	(92.26–178.3)
	MCP-1	21,723	(12,959–28,732)	20,693	(16,627–23,984)	18,618	(8496–22,436)
	MIP-1β	2863	(351.6–5706)	3781	(1342–7966)	2291	(1241–5973)
T_H_1 cytokines	IFNγ	163.1	(116.5–273.6)	251.3	(178.3–424.4)	166.7	(133.8–283.5)
	IL-2	9.75	(4.65–14.7)	11.7	(5.085–26.12)	10.82	(6.228–26.73)
	IL-12p40	229.3	(151.9–287.5)	292.5	(92.7–501)	249.6	(176.4–328.5)
	IL-12p70	106.9	(48.68–126)	110.5	(48.35–208)	97.32	(61.34–130.6)
T_H_2 cytokines	IL-4	14.66	(7.29–27.71)	19.59	(7.705–57.5)	20.34	(12.96–45.41)
	IL-5	3.3	(0.25–5.36)	2.97	(0.25–4.52)	3.42	(0.465–5.375)
	IL-13	29	(14.2–46.44)	33.33	(16.71–86.75)	26.02	(19.9–42.24)
T_H_17 cytokine	IL-17	17.04	(8.29–20.41)	20.91^**#**^	(10.73–53.02)	14.53	(9.393–21.33)
Regulatory cytokines	IL-10	1321	(654–2257)	1071	(302.4–1709)	1228	(594.4–1654)
	TGF-β	2063	(1213–3724)	1893	(911.4–3610)	2728	(1763–3831)

# p value < 0.05 when comparing AFF with HC

* p value < 0.05 when comparing AFF with NON

† p value < 0.05 when comparing NON with HC

### Cytokine and chemokine production after PHA stimulation

**Fig. 3:**
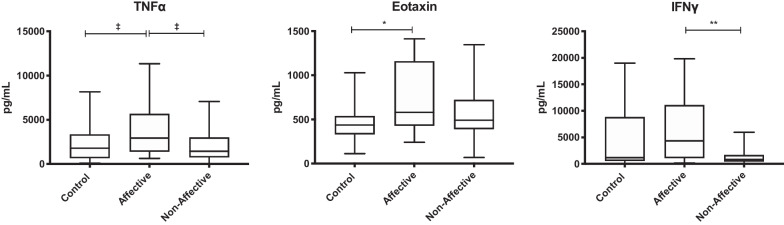
Cytokine and chemokine production following stimulation with PHA: box and whiskers plots depict median, upper and lower interquartile ranges, *p < 0.05, **p < 0.01, ‡ indicates comparisons that were significant prior to multiple testing correction. HC *n* = 45, AFF *n* = 22, NON *n* = 31

After stimulating with PHA to generate general immune activation, Kruskal–Wallis tests showed significant differences in eotaxin (*p* < 0.01), IFNγ (*p* < 0.05), and TNF-α (*p* < 0.05) across the three groups. After statistical correction for multiple comparisons, Mann–Whitney *U* tests showed that there was significantly decreased production of IFNγ (*p* = 0.0063) in the NON group compared to the AFF group. Eotaxin (*p* = 0.0084) was increased in the AFF group compared to HC (Table 4, Fig. 3). In addition, there was a trend for increased TNF-α in the AFF group compared both HC and NON groups; however, this did not reach significance after correction for multiple comparisons.

**Table 4:** Concentration of cytokines/chemokines after PHA stimulation

		HC		AFF		NON	
		Median	(IQR)	Median	(IQR)	Median	(IQR)
Growth factors	G-CSF	1073	(176.2–5540)	1742	(507.6–3131)	1568	(502.1–3992)
	GM-CSF	2052	(1068–4299)	2688	(1174–7231)	2144	(1210–4119)
	IL-7	230.8	(155.2–356.2)	212.6	(174.7–364.8)	207.8	(153.6–376.2)
Innate inflammatory cytokines	TNF-α	1788	(660–3352)	2935	(1398–5679)	1439	(744.5–3003)
	TNF-β	132.4	(45.81–249.9)	90.850	(60.01–225.2)	94.32	(52.81–259.4)
	IL-1α	255.1	(119.7–483.3)	260.3	(142.4–490.1)	147	(71.45–383.6)
	IL-1β	397	(163.2–701.5)	435.8	(294.9–924.8)	423.1	(185.2–666.8)
	IL-6	12,379	(4432–32,676)	14,652	(10,115–38,533)	14,463	(8887–29,259)
	IL-15	BLD	( -)	BLD	( -)	BLD	( -)
	IFNα2	143.5	(107.3–255.6)	169.3	(105.9–271.6)	233.9	(94.19–308.3)
Innate inflammatory chemokines	Eotaxin	437.1	(332.3–538.4)	579.6^**#**^	(427.6–1159)	491.5	(390.6–721.6)
	IL-8	58,839	(45,963–72,654)	72,251	(60,626–79,861)	62,381	(46,572–73,048)
	IP-10	1636	(737.4–2676)	674.7	(507.4–1881)	1110	(346.7–2251)
	MCP-1	45,328	(32,987–56,223)	46,623	(40,973–53,608)	42,272	(36,682–55,723)
	MIP-1β	8413	(3240–14,641)	9661	(4050–17,779)	6455	(2483–11,844)
T_H_1 cytokines	IFNγ	1173	(561.6–8849)	4341*	(1063–11,102)	851.6	(450.8–1678)
	IL-2	38.67	(19.71–88.13)	43.55	(21.72–94.29)	34.23	(22.01–85.62)
	IL-12p40	469.1	(279.1–550.6)	463.9	(314.4–679.1)	416.5	(317.4–586.8)
	IL-12p70	233.7	(122.4–339.8)	257.7	(126.7–344.4)	212.6	(117.7–255.6)
T_H_2 cytokines	IL-4	54.87	(25.72–83.06)	95.57	(37.74–124.6)	64.69	(34.19–111.5)
	IL-5	31.31	(5.283–55.71)	37.21	(12.36–66.15)	29.31	(5.013–70.01)
	IL-13	547	(189.5–1356)	920.7	(273.3–2179)	845.5	(232.2–1494)
T_H_17 cytokine	IL-17	171.8	(109.4–261.1)	225.2	(114.4–336.9)	198.8	(113–415.3)
Regulatory cytokines	IL-10	634.2	(267.4–1287)	534.4	(306.3–918.8)	547.6	(233–1017)
	TGF-β	2460	(1253–3338)	2056	(1233–4097)	2530	(1506–3467)

*BLD *below the limit of detection

# p value < 0.05 when comparing AFF with HC

* p value < 0.05 when comparing AFF with NON

† p value < 0.05 when comparing NON with HC

### Cytokine and chemokine production after α-CD3 stimulation

**Fig. 4:**
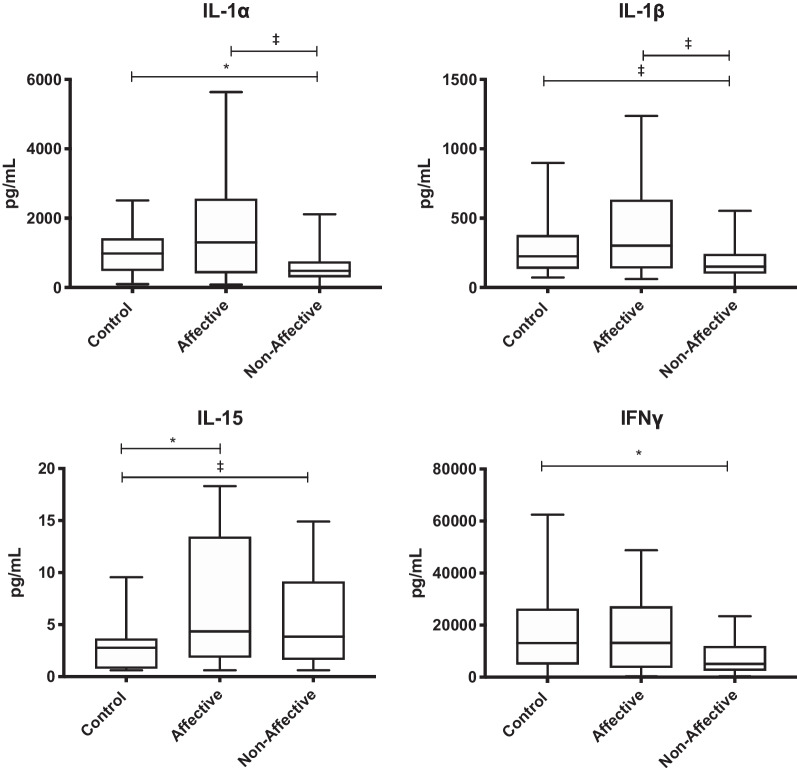
Cytokine production after stimulation with α-CD3/α-CD28: box and whiskers plots depict median, upper and lower interquartile ranges, *p < 0.05. HC *n* = 45, ‡ indicates comparisons that were significant prior to multiple testing correction. HC *n* = 45, AFF *n* = 22, NON *n* = 31

To stimulate primarily T cell responses, PBMC were activated with α-CD3/CD28. Kruskal–Wallis tests showed significant differences in IFNγ (*p* < 0.05), IL-1α (*p* < 0.05), IL-1β (*p* < 0.05), and IL-15 (*p* < 0.05) across the three groups. There was significantly increased production of IL-15 (*p* = 0.0223) in the AFF group compared to HC as assessed in pairwise comparisons with Mann–Whitney *U* tests. Moreover, IFNγ (*p* = 0.0164) and IL-1α (*p* = 0.0359) were decreased in the NON group compared to HC. There was a trend for decreased IL-1β and IL-15 production in NON compared to HC, but this did not reach statistical significance after multiple testing correction. There was also a non-significant trend for decreased IL-1α and IL-1β in the NON group compared to the AFF group (Table 5, Fig. 4) after correcting for multiple comparisons.

**Table 5:** Concentration of cytokines/chemokines after α-CD3/α-CD28 stimulation

		HC		AFF		NON	
		Median	(IQR)	Median	(IQR)	Median	(IQR)
Growth factors	G-CSF	51.58	(32.84–148.3)	122.9	(32.68–253.6)	59.57	(39.84–169.6)
	GM-CSF	3340	(1913–4759)	3576	(2269–6177)	3863	(2176–6296)
	IL-7	102.5	(75.29–144.5)	101.2	(71.69–168.7)	92.73	(65.81–134.1)
Innate inflammatory cytokines	TNF-α	4789	(2849–7405)	5777	(3372–9193)	3418	(2538–6753)
	TNF-β	513.7	(276.4–797.2)	616.000	(400.9–909)	303	(231.2–651.1)
	IL-1α	981.4	(485–1416)	1304	(412–2565)	481.9†	(297.1–753.8)
	IL-1β	225.2	(135.6–377.5)	301.7	(138.7–633.7)	150.8	(101.2–242.2)
	IL-6	2223	(1286–11,887)	1429	(878.2–2792)	1943	(966.2–4068)
	IL-15	2.78	(0.78–3.665)	4.34^**#**^	(1.83–13.45)	3.835	(1.6–9.143)
	IFNα2	50.17	(35.4–71.4)	48.33	(32.23–92.72)	45.64	(34.12–71.77)
Innate inflammatory chemokines	Eotaxin	139.5	(104.8–223.7)	178.6	(143–336.2)	149.4	(127.7–233.4)
	IL-8	14,590	(11,646–19,854)	18,971	(14,800–20,266)	15,339	(12,828–19,943)
	IP-10	1207	(418.2–2672)	843.9	(392.4–1652)	623.2	(199.4–1962)
	MCP-1	11,567	(9328–13,727)	12,029	(10,433–13,986)	11,523	(9074–13,589)
	MIP-1β	3369	(1613–7961)	3387	(1466–6958)	1792	(1114–5822)
T_H_1 cytokines	IFNγ	13,121	(4883–26,333)	13,157	(3595–27,204)	5084†	(2472–11,965)
	IL-2	101.3	(31.31–289.6)	96.61	(49.06–224.5)	57.24	(24.56–174.4)
	IL-12p40	150.2	(112.2–233.9)	150.2	(84.96–275.9)	141.3	(97.14–192.8)
	IL-12p70	73.66	(49.81–118.2)	94.27	(34.4–157.8)	67.83	(45.71–116.1)
T_H_2 cytokines	IL-4	102.8	(52.07–150)	83.22	(56.47–204.2)	75.58	(44.41–117.8)
	IL-5	84.22	(44.73–174.1)	127.5	(31.31–198.4)	86.28	(34.56–202.5)
	IL-13	1651	(1053–2626)	2080	(1109–3263)	1504	(859.3–3230)
T_H_17 cytokine	IL-17	127.5	(73.49–239.1)	167.1	(123.5–423.7)	148.6	(53.14–224.7)
Regulatory cytokines	IL-10	781.2	(480.6–1224)	588.3	(296.2–1056)	465.9	(277.5–914.1)
	TGF-β	2152	(1190–3307)	2040	(1110–3158)	2301	(1266–2874)

# p value < 0.05 when comparing AFF with HC

* p value < 0.05 when comparing AFF with NON

† p value < 0.05 when comparing NON with HC

### Spearman correlations

After correlation analysis, cytokines that were significantly different across groups at baseline were associated with increased SANS scores (Additional file 1: Table S1). These include eotaxin, GM-CSF, IFNγ, IL-1β, IL-4, IL-6, IL-12p40 and TNF-α. Correlations to baseline inflammatory cytokine production were stronger in males. Additionally, production of IL-1β and IL-17 after LPS stimulation was also correlated with SANS scores; however, this was only seen in males (Additional file 1: Table S1).

## Discussion

**Fig. 5:**
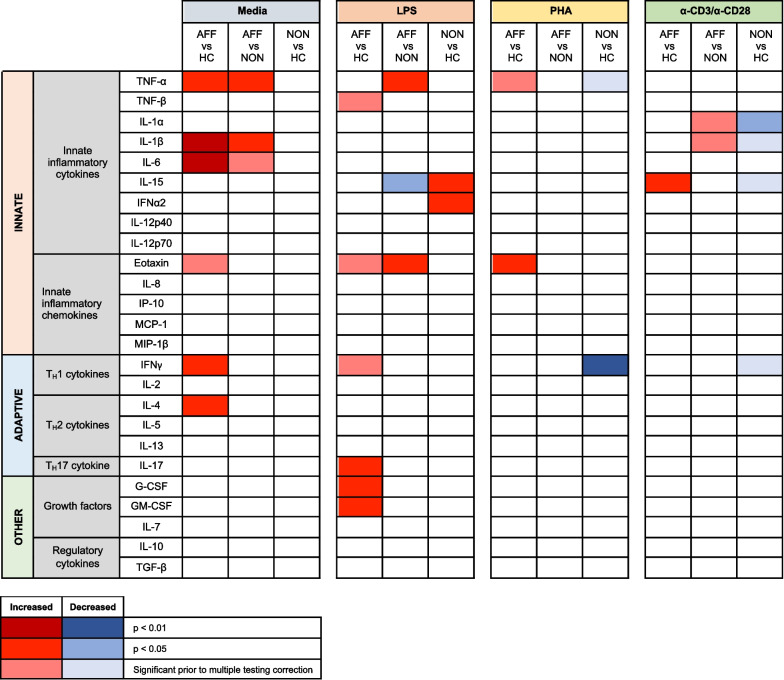
Increased and decreased cytokines across treatments and groups: heatmap plot shows group comparisons of increased (red) or decreased (blue) cytokines by stimulatory condition, with intensity of color indicating significance

To better understand functional differences of peripheral immune cells in psychotic and affective disorders, we analyzed PBMC cytokine production after cellular activation with a variety of stimuli that either triggered innate, adaptive, or general immune responses. We found differential cellular responses in the FEP patients depending on the presence or absence of an affective disorder, and when compared to controls. Specifically, we found the PBMC from AFF patients were consistently more activated under baseline and all stimulatory conditions compared to cell cultures from both the HC and NON groups (Fig. 5). Furthermore, much of this activation was associated with the innate immune response. These data are consistent with differences associated with inflammatory monocytes previously identified in psychotic and affective disorders [29, 30, 50]. The only cytokine elevated uniquely in the NON group after stimulation was IL-15, a cytokine produced by monocytes and associated with mucosal immunity. These data support functional differences in immune cells from FEP patients compared to healthy controls, as well as differences in inflammatory status of affective versus non-affective psychosis patients. These findings also support the possibility of treating affective disorders using anti-TNF-α compounds, which have shown promise in reducing depressive symptoms (reviewed in [51]).

Cytokine profiles produced by PBMC cultures without stimulation in the AFF group showed evidence of activation that may reflect responses of different cell types, including monocytes, CD4^+^ T cells, and potentially NK cells when compared to HC. More increases in cytokines related to the innate inflammatory response were seen when AFF was compared to both HC and NON cell culture baseline supernatants. These included elevated levels of IL-1β, IL-6, and TNF-α, the hallmark cytokines produced by innate immune cells in the early inflammatory immune response [52]. Eotaxin, a chemokine produced by monocytes, macrophages, and eosinophils under inflammatory conditions, was also increased in baseline conditions by AFF cells. Production of innate cytokines and chemokines can have downstream consequences on adaptive immune cell activation. Evidence of CD4^+^ T cell activation was also seen in AFF, with elevations in IFNγ and IL-4 after baseline cell culture; however, it is possible that other immune cell populations within PBMC are driving this production. IFNγ is an activator of classical inflammatory macrophages and is typically associated with T_H_1 effector cells; however, its production could also be a consequence of NK cell activation [53].

As expected, we saw evidence of innate immune activation after LPS stimulation across all groups. LPS activates TLR4 on innate immune cells such as monocytes [54]. After LPS stimulation, induced-cytokine production from cell cultures in the AFF group showed increased innate cytokines and chemokines compared to both AFF and HC groups. These data suggest that an increase in activation of innate monocytes in the AFF group is likely. There was also elevation of CD4^+^ T cell-associated cytokines IFNγ and IL-17 in the AFF group compared to HC after LPS stimulation; however, it is important to note that the PBMC fraction contains a variety of adaptive and innate cells that are all capable of producing the various cytokines, so it is possible these innate and adaptive responses are coming from mixed populations. Elevations in CD4^+^ T cell cytokines may be due to LPS activating innate cells leading to downstream responses, especially given the 48-h cell culture. NK cells can also be directly activated by LPS to produce IFNγ; however, these cells are typically associated with an antiviral or antitumor response [55]. Elevated IL-17 has previously been associated with MDD [56, 57] and suicidal behavior in BD [58]; however, findings in SCZ and non-affective psychosis are inconsistent [59–61].

IFNα2 was increased in the NON group compared to HC after LPS stimulation and trended higher in the AFF group. IFNα2 is a type I interferon secreted by virally infected cells. Type I interferons act to inhibit viral infection of other cells through altered expression of IFN-regulated genes within the infected cells, inducing an “anti-viral state” [62]. Monocytes do not typically produce IFNα2 after LPS stimulation unless they are primed with GM-CSF and IFNγ [63], both of which were elevated after LPS in the AFF group only. We previously saw increases in innate plasma cytokines in NON patients when compared to HC. In this current study, IL-15 was the only cytokine uniquely increased in the NON group compared to both AFF and HC, and this unique increase was only seen after LPS stimulation. Elevated IL-15 has been shown previously in first-episode SCZ patients, naïve to antipsychotics [64], as well as adolescents with MDD [65]; however, very few studies have included IL-15. Produced by monocytes and macrophages, IL-15 plays a role in homeostasis of NK cells and memory CD8^+^ T cells, and overexpression is associated with gastrointestinal (GI) autoimmune and inflammatory disorders [66–68]. Intestinal IL-15 has been implicated in promoting gut dysbiosis in mice and humans [69] and may be involved in predisposing individuals to celiac disease [67]. Celiac disease has been associated with SCZ for decades; however, links to mood disorders are not conclusive [70]. More research is needed here to determine if this cytokine is consistently different in NON versus AFF disorders and if there might be a link to GI dysfunction.

The observation that there were increased cytokine responses in the AFF group continued with stimulations with either PHA or α-CD3/α-CD28. Increased eotaxin was seen in the AFF group after PHA. This chemokine had also been increased in the AFF group after LPS stimulation. Eotaxin is an eosinophil-specific chemoattractant that induces the migration of eosinophils. Its production is induced by TNF-α [71], which was also elevated in the AFF group. Eotaxin is associated with asthma and allergic inflammation, and is elevated in neuropsychiatric disorders, often correlating with severity of disease; however, distinct differences between affective and psychotic disorders have not previously been shown until now [72]. After lymphocyte and T cell-specific activation with α-CD3/α-CD28, significant decreases in IFNγ production were seen in cell cultures from the NON group compared to either AFF or HC, which may suggest a dampened CD4^+^ T cell T_H_1 response in this group. The reason for this decreased response is unknown; however, it could be due in part to specific antipsychotics known to decrease IFNγ [73]. Analysis of CPZ equivalents showed no differences. In addition, we found no evidence that specific medications altered the responses (data not shown). This was similar to our previous findings when investigating the potential confounding influence of specific medication on immune responses in FEP [18, 46].

Cytokines and chemokines provide cellular communication during immune responses and are critical for recruitment, coordination, and activation of immune cells. Within the brain, they are particularly important for neurodevelopment as they are involved in induction and regulation of neurogenesis [74]. Cytokines such as IL-1β and TNF-α are constitutively expressed by glial cells within the central nervous system and play important roles in homeostasis, synapse formation, and plasticity. Under inflammatory conditions, when microglia and astroglia are activated, increased production of inflammatory cytokines can inhibit plasticity, such as long-term potentiation (LTP), and can alter LTP maintenance [75]. TNF-α upregulates IL-15 and its receptors in brain endothelial cells, and IL-15 has been found to activate NF-κB in these cells, potentially mediating permeability of the blood–brain barrier [76]. Although not direct evidence of neuroinflammation, increases in cytokine production at baseline may reflect increased steady-state inflammatory profiles that can lead to activation of immune cells in the brain and CNS and lead to pathological behavior. Our findings that baseline concentrations of several inflammatory cytokines were associated with SANS scores highlight this possibility [51]; however, further research is needed to verify these that increased baseline activation of immune cells is linked to worsening behavior in first-episode psychosis.

Dysfunctional immune regulation is an alternative hypothesis driving aberrant activation of immune cells in both the periphery and the brain. TGF-β and regulatory T cells (T_regs_) play a crucial role in appropriate immune responses and immune homeostasis. Although evidence of T_regs_ dysfunction is conflicting in psychotic disorders, increased T_regs_ were found to be associated with better clinical presentation, and lower T_regs_ were associated with worsening symptoms [77]. Reduced T_regs_ and associated TGF-β are also seen in other neurodevelopmental disorders, including autism spectrum disorder, in which immune dysfunction has also been implicated [78]. Altered T_regs_ could have implications for neuroinflammation and might explain the conflicting PET studies showing reduced radioligand binding to TSPO, suggesting reduced microglial activation. These findings, combined with increased gene expression associated with activated astrocytes in the brain, could be driving aberrant activation of microglia and altering synaptic pruning [36].

Our study had several limitations, including a small sample size. Patients were assessed and blood was drawn as close to the first episode as possible and at most within a year of diagnosis; however, the blood draws were not necessarily during the first episode. We found no differences in the data based on length of time between inclusion in the study and when the blood draw was taken, suggesting findings are stable across that interval. However, this could be a potential limitation in this and other studies. If immune status changes with the state of the disorder, inflammation may have been missed in some patients. This study looked at PBMC, which are mixed cultures of monocytes, dendritic cells, NK cells, and T and B lymphocytes. As such, it is difficult to determine the precise source of cytokines, and there is a multi-directional influence of the individual cell subsets within the cultures. Activation of cells with different stimulants helps us overcome some of these limitations by inducing specific responses and helps tease apart some of the processes. However, our data suggest that assays looking at specific subsets are warranted. In addition, the 48-h timepoint does not provide insight into immediate immune responses. Another limitation of our study is that antipsychotics can influence inflammation and immune function [64, 79, 80] and have been seen to influence levels of IFNγ and IL-4 in stimulated PBMC from FEP patients [73]. However, the effects of antipsychotic medication are not always a consistent phenomenon [81]. We did not find any differences based on specific medications. Despite these shortcomings, our study is strengthened by utilizing stimulated cells for the purpose of identifying functional differences rather than circulating plasma or sera cytokines that can be influenced by many different environmental factors such as time of blood draw, fasting of draw, length of processing time and cross-sectional plasma/sera results can be highly heterogenous and provide limited information.

In conclusion, we found evidence of increased activation of immune cells following stimulation in FEP patients with affective disorder compared to both healthy controls and FEP patients without affective disorder. We also found that increases in baseline activation of immune cells correlated with SANS scores. Immune dysfunction is prominent in psychotic and affective disorders; however, few previous studies have attempted to delineate differences in immune cellular function in affective versus non-affective psychosis. Our findings provide potential evidence that immune dysfunction may be associated with the presence of affective disorder and that this differs from dysfunction in primary psychotic disorders. Future work should include isolating and stimulating individual cell subsets associated with the differential immune activation seen in AFF versus NON to identify which cell types are most responsible for the differences seen in this study, ideally in antipsychotic naïve FEP patients, to eliminate the confounding drug issues.

## Supplementary Information

**Additional file 1.** **Supplemental Table 1:** Spearman's correlation. Baseline and LPS-induced cytokine production correlated with SANS scores. Correlations were stronger in males.

## Data Availability

The datasets used and/or analyzed during the current study are available from the corresponding author on reasonable request.

## References

[1] Ferrari AJ, Saha S, McGrath JJ, Norman R, Baxter AJ, Vos T (2012). Health states for schizophrenia and bipolar disorder within the Global Burden of Disease 2010 Study. Popul Health Metr.

[2] Reddy MS (2010). Depression: the disorder and the burden. Indian J Psychol Med.

[3] Vigo D, Thornicroft G, Atun R (2016). Estimating the true global burden of mental illness. Lancet Psychiatry.

[4] Notter T, Coughlin JM, Gschwind T, Weber-Stadlbauer U, Wang Y, Kassiou M (2017). Translational evaluation of translocator protein as a marker of neuroinflammation in schizophrenia. Mol Psychiatry.

[5] Pedersen MS, Benros ME, Agerbo E, Børglum AD, Mortensen PB (2012). Schizophrenia in patients with atopic disorders with particular emphasis on asthma: A Danish population-based study. Schizophr Res.

[6] Benros ME, Waltoft BL, Nordentoft M, Ostergaard SD, Eaton WW, Krogh J (2013). Autoimmune diseases and severe infections as risk factors for mood disorders: a nationwide study. JAMA Psychiat.

[7] Machón RA, Mednick SA, Huttunen MO (1997). Adult major affective disorder after prenatal exposure to an influenza epidemic. Arch Gen Psychiatry.

[8] Brown AS, Derkits EJ (2010). Prenatal infection and schizophrenia: a review of epidemiologic and translational studies. Am J Psychiatry.

[9] Köhler O, Petersen L, Mors O, Mortensen PB, Yolken RH, Gasse C (2017). Infections and exposure to anti-infective agents and the risk of severe mental disorders: a nationwide study. Acta Psychiatr Scand.

[10] Blomström Å, Karlsson H, Svensson A, Frisell T, Lee BK, Dal H (2014). Hospital admission with infection during childhood and risk for psychotic illness–a population-based cohort study. Schizophr Bull.

[11] Smith RS, Maes M (1995). The macrophage-T-lymphocyte theory of schizophrenia: additional evidence. Med Hypotheses.

[12] Smith RS (1991). The macrophage theory of depression. Med Hypotheses.

[13] Drexhage RC, Knijff EM, Padmos RC, Heul-Nieuwenhuijzen L, Beumer W, Versnel MA (2010). The mononuclear phagocyte system and its cytokine inflammatory networks in schizophrenia and bipolar disorder. Expert Rev Neurother.

[14] Upthegrove R, Manzanares-Teson N, Barnes NM (2014). Cytokine function in medication-naive first episode psychosis: A systematic review and meta-analysis. Schizophr Res.

[15] Pillinger T, Osimo EF, Brugger S, Mondelli V, McCutcheon RA, Howes OD (2018). A Meta-analysis of Immune Parameters, Variability, and Assessment of Modal Distribution in Psychosis and Test of the Immune Subgroup Hypothesis. Schizophr Bull.

[16] Munkholm K, Vinberg M, Vedel KL (2013). Cytokines in bipolar disorder: a systematic review and meta-analysis. J Affect Disord.

[17] Modabbernia A, Taslimi S, Brietzke E, Ashrafi M (2013). Cytokine alterations in bipolar disorder: a meta-analysis of 30 studies. Biol Psychiatry.

[18] Lesh TA, Careaga M, Rose DR, McAllister AK, Van Water J, Carter CS (2018). Cytokine alterations in first-episode schizophrenia and bipolar disorder: relationships to brain structure and symptoms. J Neuroinflam.

[19] Goldsmith DR, Rapaport MH, Miller BJ (2016). A meta-analysis of blood cytokine network alterations in psychiatric patients: comparisons between schizophrenia, bipolar disorder and depression. Mol Psychiatry.

[20] Iwasaki A, Medzhitov R (2015). Control of adaptive immunity by the innate immune system. Nat Immunol.

[21] Gruys E, Toussaint MJM, Niewold TA, Koopmans SJ (2005). Acute phase reaction and acute phase proteins. J Zhejiang Univ Sci B.

[22] Dowlati Y, Herrmann N, Swardfager W, Liu H, Sham L, Reim EK (2010). A meta-analysis of cytokines in major depression. Biol Psychiatry.

[23] Miller BJ, Buckley P, Seabolt W, Mellor A, Kirkpatrick B (2011). Meta-analysis of cytokine alterations in schizophrenia: clinical status and antipsychotic effects. Biol Psychiatry.

[24] Mazza MG, Lucchi S, Rossetti A, Clerici M (2019). Neutrophil-lymphocyte ratio, monocyte-lymphocyte ratio and platelet-lymphocyte ratio in non-affective psychosis: A meta-analysis and systematic review. World J Biol Psychiatry..

[25] Orhan F, Schwieler L, Fatouros-Bergman H, Malmqvist A, Cervenka S, Collste K (2018). Increased number of monocytes and plasma levels of MCP-1 and YKL-40 in first-episode psychosis. Acta Psychiatr Scand.

[26] Barbosa IG, Rocha NP, Assis F, Vieira ÉLM, Soares JC, Bauer ME (2014). Monocyte and lymphocyte activation in bipolar disorder: a new piece in the puzzle of immune dysfunction in mood disorders. Int J Neuropsychopharmacol.

[27] Drexhage RC, Hoogenboezem TA, Cohen D, Versnel MA, Nolen WA, van Beveren NJ (2011). An activated set point of T-cell and monocyte inflammatory networks in recent-onset schizophrenia patients involves both pro- and anti-inflammatory forces. Int J Neuropsychopharmacol.

[28] Breunis MN, Kupka RW, Nolen WA, Suppes T, Denicoff KD, Leverich GS (2003). High numbers of circulating activated T cells and raised levels of serum IL-2 receptor in bipolar disorder. Biol Psychiatry.

[29] Drexhage RC, van der Heul-Nieuwenhuijsen L, Padmos RC, van Beveren N, Cohen D, Versnel MA (2010). Inflammatory gene expression in monocytes of patients with schizophrenia: overlap and difference with bipolar disorder: A study in naturalistically treated patients. Int J Neuropsychopharmacol.

[30] Carvalho LA, Bergink V, Sumaski L, Wijkhuijs J, Hoogendijk WJ, Birkenhager TK (2014). Inflammatory activation is associated with a reduced glucocorticoid receptor alpha/beta expression ratio in monocytes of inpatients with melancholic major depressive disorder. Transl Psychiatry.

[31] Becking K, Haarman BCM, van der Lek RFR, Grosse L, Nolen WA, Claes S (2015). Inflammatory monocyte gene expression: trait or state marker in bipolar disorder?. Int J Bipolar Dis..

[32] Grosse L, Carvalho LA, Wijkhuijs AJM, Bellingrath S, Ruland T, Ambrée O (2015). Clinical characteristics of inflammation-associated depression: Monocyte gene expression is age-related in major depressive disorder. Brain Behav Immun.

[33] Kim Y-K, Jung H-G, Myint A-M, Kim H, Park S-H (2007). Imbalance between pro-inflammatory and anti-inflammatory cytokines in bipolar disorder. J Affect Disord.

[34] Grosse L, Hoogenboezem T, Ambrée O, Bellingrath S, Jörgens S, de Wit HJ (2016). Deficiencies of the T and natural killer cell system in major depressive disorder. T regulatory cell defects are associated with inflammatory monocyte activation. Brain Behavior Immunity..

[35] Wang AK, Miller BJ (2018). Meta-analysis of cerebrospinal fluid cytokine and tryptophan catabolite alterations in psychiatric patients: comparisons between schizophrenia, bipolar disorder, and depression. Schizophr Bull.

[36] Corsi-Zuelli F, Deakin B (2021). Impaired regulatory T cell control of astroglial overdrive and microglial pruning in schizophrenia. Neurosci Biobehav Rev.

[37] Hughes HK, Ashwood P (2020). Overlapping evidence of innate immune dysfunction in psychotic and affective disorders. Brain Behav Immunity Health.

[38] Steiner J, Bielau H, Brisch R, Danos P, Ullrich O, Mawrin C (2008). Immunological aspects in the neurobiology of suicide: Elevated microglial density in schizophrenia and depression is associated with suicide. J Psychiatr Res.

[39] Torres-Platas SG, Cruceanu C, Chen GG, Turecki G, Mechawar N (2014). Evidence for increased microglial priming and macrophage recruitment in the dorsal anterior cingulate white matter of depressed suicides. Brain Behav Immun.

[40] Schnieder TP, Trencevska I, Rosoklija G, Stankov A, Mann JJ, Smiley J (2014). Microglia of prefrontal white matter in suicide. J Neuropathol Exp Neurol.

[41] Holmes SE, Hinz R, Conen S, Gregory CJ, Matthews JC, Anton-Rodriguez JM (2018). Elevated translocator protein in anterior cingulate in major depression and a role for inflammation in suicidal thinking: a positron emission tomography study. Biol Psychiatry.

[42] Busse S, Busse M, Schiltz K, Bielau H, Gos T, Brisch R (2012). Different distribution patterns of lymphocytes and microglia in the hippocampus of patients with residual versus paranoid schizophrenia: further evidence for disease course-related immune alterations?. Brain Behav Immun.

[43] Kim JS, Baek JH, Choi JS, Lee D, Kwon JS, Hong KS (2011). Diagnostic stability of first-episode psychosis and predictors of diagnostic shift from non-affective psychosis to bipolar disorder: A retrospective evaluation after recurrence. Psychiatry Res.

[44] Eaton WW, Pedersen MG, Nielsen PR, Mortensen PB (2010). Autoimmune diseases, bipolar disorder, and non-affective psychosis. Bipolar Disord.

[45] Noto C, Ota VK, Santoro ML, Ortiz BB, Rizzo LB, Higuchi CH (2015). Effects of depression on the cytokine profile in drug naïve first-episode psychosis. Schizophr Res.

[46] Hughes HK, Mills-Ko E, Yang H, Lesh TA, Carter CS, Ashwood P (2021). Differential macrophage responses in affective versus non-affective first-episode psychosis patients. Front Cell Neurosci.

[47] Andreasen NC (1983). The Scale for the Assessment of Negative Symptoms (SANS).

[48] Andreasen NC (1984). The Scale for the Assessment of Positive Symptoms (SAPS).

[49] Hall RC (1995). Global assessment of functioning. A modified scale. Psychosomatics.

[50] Brambilla P, Bellani M, Isola M, Bergami A, Marinelli V, Dusi N (2014). Increased M1/decreased M2 signature and signs of Th1/Th2 shift in chronic patients with bipolar disorder, but not in those with schizophrenia. Transl Psychiatry.

[51] Uzzan S, Azab AN (2021). Anti-TNF-α compounds as a treatment for depression. Molecules.

[52] Geissmann F, Manz MG, Jung S, Sieweke MH, Merad M, Ley K (2010). Development of monocytes, macrophages, and dendritic cells. Science (New York, NY).

[53] Schoenborn JR, Wilson CB (2007). Regulation of interferon-γ during innate and adaptive immune responses. Adv Immunol.

[54] Kawasaki T, Kawai T (2014). Toll-like receptor signaling pathways. Front Immunol.

[55] Kanevskiy LM, Erokhina SA, Streltsova MA, Ziganshin RH, Telford WG, Sapozhnikov AM (2019). The Role of O-Antigen in LPS-Induced Activation of Human NK Cells. J Immunol Res.

[56] Davami MH, Baharlou R, Ahmadi Vasmehjani A, Ghanizadeh A, Keshtkar M, Dezhkam I (2016). Elevated IL-17 and TGF-β Serum Levels: A Positive Correlation between T-helper 17 cell-related pro-inflammatory responses with major depressive disorder. Basic Clin Neurosci.

[57] Saraykar S, Cao B, Barroso LS, Pereira KS, Bertola L, Nicolau M (2018). Plasma IL-17A levels in patients with late-life depression. Braz J Psychiatry.

[58] Keshri N, Nandeesha H, Kattimani S (2018). Elevated interleukin-17 and reduced testosterone in bipolar disorder Relation with suicidal behaviour. Asian J Psychiatry..

[59] Borovcanin M, Jovanovic I, Radosavljevic G, Djukic Dejanovic S, Bankovic D, Arsenijevic N (2012). Elevated serum level of type-2 cytokine and low IL-17 in first episode psychosis and schizophrenia in relapse. J Psychiatr Res.

[60] Dimitrov DH, Lee S, Yantis J, Valdez C, Paredes RM, Braida N (2013). Differential correlations between inflammatory cytokines and psychopathology in veterans with schizophrenia: Potential role for IL-17 pathway. Schizophr Res.

[61] Fang X, Zhang Y, Fan W, Tang W, Zhang C (2018). Interleukin-17 alteration in first-episode psychosis: a meta-analysis. Mol Neuropsychiatry.

[62] Landolfo S, De Andrea M, Ratcliffe MJH (2016). Interferon α/β. Encyclopedia of Immunobiology.

[63] Hayes MP, Enterline JC, Gerrard TL, Zoon KC (1991). Regulation of interferon production by human monocytes: requirements for priming for lipopolysaccharide-induced production. J Leukoc Biol.

[64] de Witte L, Tomasik J, Schwarz E, Guest PC, Rahmoune H, Kahn RS (2014). Cytokine alterations in first-episode schizophrenia patients before and after antipsychotic treatment. Schizophr Res.

[65] Pérez-Sánchez G, Becerril-Villanueva E, Arreola R, Martínez-Levy G, Hernández-Gutiérrez ME, Velasco-Velásquez MA (2018). Inflammatory profiles in depressed adolescents treated with fluoxetine: an 8-week follow-up open study. Mediators Inflamm.

[66] Pagliari D, Cianci R, Frosali S, Landolfi R, Cammarota G, Newton EE (2013). The role of IL-15 in gastrointestinal diseases: A bridge between innate and adaptive immune response. Cytokine Growth Factor Rev.

[67] Abadie V, Jabri B (2014). IL-15: a central regulator of celiac disease immunopathology. Immunol Rev.

[68] van Heel DA (2006). Interleukin 15: its role in intestinal inflammation. Gut.

[69] Meisel M, Mayassi T, Fehlner-Peach H, Koval JC, O'Brien SL, Hinterleitner R (2017). Interleukin-15 promotes intestinal dysbiosis with butyrate deficiency associated with increased susceptibility to colitis. ISME J.

[70] Porcelli B, Verdino V, Bossini L, Terzuoli L, Fagiolini A (2014). Celiac and non-celiac gluten sensitivity: a review on the association with schizophrenia and mood disorders. Auto Immun Highlights.

[71] Rothenberg ME (1999). Eotaxin. An essential mediator of eosinophil trafficking into mucosal tissues. Am J Respir Cell Mol Biol.

[72] Teixeira AL, Gama CS, Rocha NP, Teixeira MM (2018). Revisiting the Role of Eotaxin-1/CCL11 in Psychiatric Disorders. Front Psychiatry..

[73] Al-Amin MM, Nasir Uddin MM, Mahmud RH (2013). Effects of antipsychotics on the inflammatory response system of patients with schizophrenia in peripheral blood mononuclear cell cultures. Clin Psychopharmacol Neurosci.

[74] Deverman BE, Patterson PH (2009). Cytokines and CNS development. Neuron.

[75] Rizzo FR, Musella A, De Vito F, Fresegna D, Bullitta S, Vanni V (2018). Tumor necrosis factor and interleukin-1β modulate synaptic plasticity during neuroinflammation. Neural Plast.

[76] Stone KP, Kastin AJ, Pan W (2011). NFĸB is an unexpected major mediator of interleukin-15 signaling in cerebral endothelia. Cell Physiol Biochem.

[77] Corsi-Zuelli F, Deakin B, de Lima MHF, Qureshi O, Barnes NM, Upthegrove R (2021). T regulatory cells as a potential therapeutic target in psychosis? Current challenges and future perspectives. Brain Behav Immun Health.

[78] Hughes HK, Mills Ko E, Rose D, Ashwood P (2018). Immune dysfunction and autoimmunity as pathological mechanisms in autism spectrum disorders. Front Cell Neurosci.

[79] Kubistova A, Horacek J, Novak T (2012). Increased interleukin-6 and tumor necrosis factor alpha in first episode schizophrenia patients versus healthy controls. Psychiatr Danub.

[80] MacDowell KS, García-Bueno B, Madrigal JLM, Parellada M, Arango C, Micó JA (2013). Risperidone normalizes increased inflammatory parameters and restores anti-inflammatory pathways in a model of neuroinflammation. Int J Neuropsychopharmacol.

[81] Theodoropoulou S, Spanakos G, Baxevanis CN, Economou M, Gritzapis AD, Papamichail MP (2001). Cytokine serum levels, autologous mixed lymphocyte reaction and surface marker analysis in never medicated and chronically medicated schizophrenic patients. Schizophr Res.

